# Mechanisms of Pharmaceutical Therapy and Drug Resistance in Esophageal Cancer

**DOI:** 10.3389/fcell.2021.612451

**Published:** 2021-02-11

**Authors:** Chengyi Mao, Xiaoxi Zeng, Chao Zhang, Yushang Yang, Xin Xiao, Siyuan Luan, Yonggang Zhang, Yong Yuan

**Affiliations:** ^1^Department of Thoracic Surgery West China Hospital, Sichuan University, Chengdu, China; ^2^West China Biomedical Big Data Center, West China Hospital, Sichuan University, Chengdu, China; ^3^Department of Periodical Press, National Clinical Research Center for Geriatrics, West China Hospital, Sichuan University, Chengdu, China; ^4^Nursing Key Laboratory of Sichuan Province, Chengdu, China; ^5^Chinese Evidence-Based Medicine Center, West China Hospital, Sichuan University, Chengdu, China

**Keywords:** esophageal cancer, chemotherapy, molecular targeted therapy, immunotherapy, drug resistance

## Abstract

Pharmaceutical therapies are essential for esophageal cancer (EC). For the advanced EC, the neoadjuvant therapy regimen, including chemotherapy plus radiotherapy and/or immunotherapy, is effective to achieve clinical benefit, even pathological complete response. For the unresectable, recurrent, and metastatic EC, the pharmaceutical therapy is the limited effective regimen to alleviate the disease and prolong the progression-free survival and overall survival. In this review, we focus on the pharmaceutical applications in EC treatment including cytotoxic agents, molecular targeted antibodies, and immune checkpoint inhibitors (ICIs). The chemotherapy regimen is based on cytotoxic agents such as platinum-based complexes, fluorinated pyrimidines and taxenes. Although the cytotoxic agents have been developed in past decades, the standard chemotherapy regimen is still the cisplatin and 5-FU or paclitaxel because the derived drugs have no significant advantages of overcoming the shortcomings of side effects and drug resistance. The targeted molecular therapy is an essential supplement for chemotherapy; however, there are only a few targeted therapies available in clinical practice. Trastuzumab and ramucirumab are the only two molecular therapy drugs which are approved by the US Food and Drug Administration to treat advanced and/or metastatic EC. Although the targeted therapy usually achieves effective benefits in the early stage therapy of EC, the patients will always develop drug resistance during treatment. ICIs have had a significant impact on routine clinical practice in cancer treatment. The anti-programmed cell death-1 monoclonal antibodies pembrolizumab and nivolumab, as the ICIs, are recommended for advanced EC by several clinical trials. However, the significant issues of pharmaceutical treatment are still the dose-limiting side effects and primary or secondary drug resistance. These defects of pharmaceutical therapy restrain the clinical application and diminish the effectiveness of treatment.

## Introduction

Esophageal cancer (EC) is a global health challenge. It ranks the seventh most common cancer incidence and the sixth most common cause of cancer-related death worldwide (Li et al., 2019; Zhang et al., 2020). The histologic types of EC are mostly composed of esophageal squamous cell carcinoma (ESCC) and esophageal adenocarcinoma (EAC), which have distinct epidemiology and biology. ESCC are highly prevalent in the East, East Africa and South America and account for 90% of EC. EAC is more common in Western developed countries than that in developing countries (Smyth et al., 2017; Chen et al., 2019).

The treatment regimen for EC is dependent on the general status of patient and the tumor stage, mainly the TNM stage (Lordick et al., 2016). Patients with early stage tumors should be treated with endoscopic or surgical resection, whereas local advanced tumors should be treated with systemic treatment regimen. Patients that are not suitable for surgical treatment should be treated with systemic regimens including definitive chemoradiotherapy, targeted therapy, immunotherapy, and palliative treatment (Smyth et al., 2017).

Most patients seek medical attention because of a period of progressive dysphagia (Lagergren et al., 2017), therefore, radical surgery is possible in only 15–20% of all cases (Zhao et al., 2020). Moreover, for resectable locally advanced EC, the overall prognosis is poor with surgery alone (Gebski et al., 2007). For the advanced or metastasis EC, although combination therapy has prolonged overall survival, the current median survival time remains almost 1 year (Shah et al., 2017; Ho and Smyth, 2020).

Although the overall survival of EC has improved in the past decades because of medicine development (Ho and Smyth, 2020), the overall 5-year survival rate is still 20% (Zeng et al., 2015; Njei et al., 2016). The main causes of treatment failure are recurrence and metastasis concurrent with treatment resistance. In this review, we focus on the most common pharmaceutical applications for EC and its drug resistance including chemotherapy, molecular targeted therapy, and immunotherapy.

## Chemotherapy

For EAC, the FLOT regimen including docetaxel, oxaliplatin, leucovorin, and fluorouracil is recognized as the standard perioperative chemotherapy regimen. The regimen including cisplatin and fluorouracil is the alternative treatment while the FLOT regimen should not be implemented (Shah et al., 2020). For ESCC, the cisplatin plus fluorouracil regimen is the standard chemotherapy (Chen et al., 2019). It can be seen that the platinum containing cytotoxic agents are still the standard regimen of chemotherapy for EC.

## Platinum Complexes

Cisplatin or *cis*-dichloro-diammine-platinum (II) is the representative drug of platinum complex. It was first used to a cancer patient in 1971 (Lebwohl and Canetta, 1998). Since then, platinum-based cytotoxic agents are widely used in tumor treatment (Shimizu et al., 2020). However, the mechanisms that determine sensitivity and resistance to platinum drugs remain elusive (De Vries et al., 2020).

The antitumor activities of cisplatin are associated with the formation of certain kinetically stable cisplatin–DNA adducts and many platinum-based drugs have been designed and synthesized based on the classical structure-activity relationships (Yang and Wang, 1996; Wheate et al., 2010). The general platinum-based complexes (II) structure has been summarized as [PtA_2_X_2_] and platinum-based complexes (IV) structure has been summarized as [PtA_2_X_2_Y_2_]. In these chemical structures, the Pt means metal platinum, the A_2_ mean two monodentate or one bidentate amine ligand, the X_2_ mean two monodentate or one bidentate anionic ligand and the Y means hydroxo, chloro, or carboxylato (Galanski, 2006).

The formation of intra-strand crosslink adducts leads to an impairment of DNA expression and stability (Yang and Wang, 1996; Heringova et al., 2009). These lesions could cause mutations and have detrimental effects on replication and transcription, which are thought to be the crucial antitumor activity of platinum-based drugs and ultimately lead to cellular apoptosis (Bai L. et al., 2017). Furthermore, cisplatin is known to interfere with cellular RNA processing by binding to RNA and assisting the antitumor activity of the drug (Chapman and Derose, 2010).

The platinum-based drug not only impairs cancerous cells but also the normal cells (Kelland, 2007). The toxicity of platinum-based drugs is directly associated with the aquated of the leaving groups (Galluzzi et al., 2012) and nephrotoxicity, neurotoxicity, ototoxicity, and myelosuppression are the most common side effects (Piccart et al., 2001). In order to diminish the toxicity and side effects of cisplatin, pharmacologists are dedicated to discovering new leaving groups such as carboplatin, oxaliplatin, nedaplatin, lobaplatin, and heptaplatin (Wheate et al., 2010; Dilruba and Kalayda, 2016; Bai L. et al., 2017). However, the toxicity and side effects are still the causes of dosage range limiting in clinic, such as myelosuppression of carboplatin, neurotoxicity and gastrointestinal toxicity of oxaliplatin (Hanada et al., 2008; Zhong et al., 2020), none of the newly platinum-based analogs have a more comprehensive effect than the cisplatin (Najjar et al., 2017). Because these new platinum-based drugs are derived from a basic cisplatin structure, the defects of cisplatin are inherited. Therefore, cisplatin is still the most widely used platinum drug in clinic, and there are only three agents including cisplatin, carboplatin, and oxaliplatin which have become globally used. Another three agents including nedaplatin, lobaplatin, and heptaplatin have received approval in regional areas (Wheate et al., 2010; Dilruba and Kalayda, 2016; Bai L. et al., 2017).

## Resistance in Platinum-Based Drugs

Beyond the side effects of platinum-based drugs diminishing the effectiveness of clinical practice, the resistance of them, including intrinsic or acquired resistance, also limits the clinical application. Furthermore, the severe side effects of cisplatin restrict the dosage intake and the dose delivered to patients can be sub-lethal to tumors, which means that it could develop resistance in further treatment.

**FIGURE 1:**
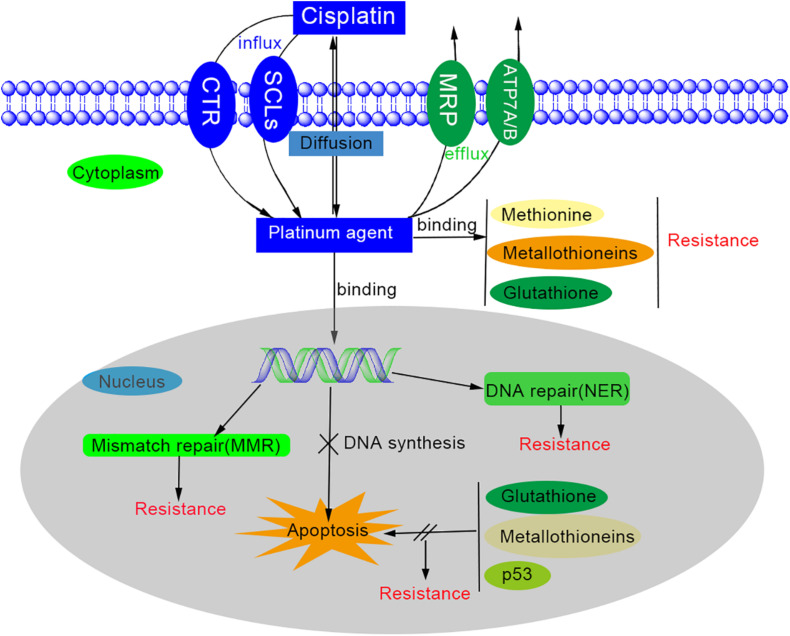
Schematic representation of drug effect and resistance of cisplatin.

However, the underlying mechanisms are still far from elucidated. The main mechanisms of platinum-based drug resistance are possibly associated with changed cellular platinum accumulation, increased detoxification system, increased DNA repair, decreased apoptosis, and autophagy (Figure 1) (Kehe and Szinicz, 2005; Wheate et al., 2010; Zhou et al., 2020).

First, to accumulate the platinum antitumor agents inside the cells is the necessary process for cytotoxicity, so platinum resistance would generate while the platinum agent influx decreased and/or efflux increased. The way that platinum enters the cell is thought to be passive diffusion and through gated channels (Gately and Howell, 1993; Puckett et al., 2010). There are multiple transporters involved in platinum influx/efflux (Zhou et al., 2020), such as solute carrier superfamily (SLCs) of membrane transporters (Perland and Fredriksson, 2017), copper transporter 1/2(CTR1/2) (Holzer and Howell, 2006), copper-transporting ATPases (ATP 7A/7B) (Gupta and Lutsenko, 2009), multidrug resistance protein subfamily (MPR) (Yaneff et al., 2019) etc. The organic cation transporters and copper transporter are related to the influx, while ATP 7A/7B and MPR2 are involved in the isolation and efflux of platinum agents (Zhou et al., 2020). However, the mechanism of uptake of platinum-based drugs is not elucidated (Hall et al., 2008). Secondly, platinum agents can be deactivated by binding to detoxification components such as glutathione (GSH), methionine, metallothioneins, and other cysteine-rich proteins. This binding depletes cytoplasmic antioxidant reserves and results in the oxidative stress in cells. On the other hand, while the cytoplasmic nucleophiles level is elevated, the available reactive cisplatin would be diminished and thus contribute to cisplatin resistance (Dilruba and Kalayda, 2016; Zhou et al., 2020). Thirdly, the DNA repair process is significantly increased in platinum resistance cells (Wynne et al., 2007). Although platinum-based agents can induce cytotoxicity by forming the platinum-DNA adducts, the DNA lesion could be repaired by the DNA repair process (Zhou et al., 2020). One of these DNA repair processes is nucleotide excision repair (NER) system, which can remove most intra-strand crosslinks through reconstituted genetic integrity by excising damaged nucleotides and synthesizing DNA (Roos and Kaina, 2013). The expression level of excision repair cross-complementing (ERCC) members and breast cancer susceptibility genes (BRCAs) also have significant influence on platinum resistance (Dann et al., 2012; Foulkes and Shuen, 2013; Muggia and Safra, 2014). Fourthly, the dysfunction of apoptosis may be one of the causes of platinum drug resistance. The apoptosis would be activated while the DNA repair fails or excessive DNA lesions occurs after platinum agents and mitochondria will generate surplus reactive oxygen species (ROS) to kill the cells. However, this reaction may be neutralized by glutathione and metallothioneins. The platinum-resistant cells usually have a higher threshold to trigger apoptosis due to the defection of mitochondrial signaling and the overexpression of anti-apoptotic proteins. Many factors contribute to the regulation of apoptosis, including the signal pathways (such as MAPK/ERK, PI3k/AKT, NF-*k*B, Nrf2, p53), the tumor microenvironment (TME) (including hypoxia-inducible factor, HIF), cancer-associated fibroblasts (CAFs), and epigenetic regulation (Ramadoss et al., 2017; Zhou et al., 2020). Last but not least, autophagy was observed to be increased in platinum-resistant cells after platinum-based drug treatment (Wang Z. et al., 2019). Autophagy is a self-digestion process and essential for nutrient regulation, intracellular quality control and homeostasis (Mizushima and Klionsky, 2007). If persistent or excessive autophagy is carried out, it will trigger cell death. When autophagy activity is inhibited by autophagy inhibitors, interference of regulatory elements, or non-coding RNAs, it has been proven to diminish platinum resistance (Zhou et al., 2020).

However, the mechanisms of platinum resistance are far from elucidated and the dose-liming side effects and cytotoxicity still hinder clinical application. Therefore, the chemotherapy is mostly concurrent with two to three cytotoxic agents to reduce dose-limiting side effects and toxicity of platinum complexes. The most common concurrent cytotoxic agents in EC are fluorinated pyrimidines (5-fluorouracil) and taxanes (paclitaxel or docetaxel).

## Fluorinated Pyrimidines

5-Fluorouracil (5-FU) is the representative drug of fluorinated pyrimidine, the chemical formula of which is C_4_H_3_FN_2_O_2_. It was first synthesized as the antitumor agent in 1957 by modified a pyrimidine chemical uracil, in which the hydrogen at the carbon-5 position of the pyrimidine ring was replaced by a fluorine atom (Alvarez et al., 2012).

The biological activities of 5-FU including antitumor activity and systemic toxicities remain only partly characterized. A primary theory is the lethal synthesis in which a biological metabolism can transform a relatively non-toxic metabolite into a more toxic form (Weckbecker, 1991). 5-FU interferes with DNA synthesis and mRNA translation by being misincorporated into RNA and DNA in place of uracil or thymine, that lead to cytotoxicity and cell death (Noordhuis et al., 2004; An et al., 2007). While 5-FU enters the human body, most of it is inactivated by dihydropyrimidine dehydrogenase (DPD), and further converted to fluorodeoxyuridine monophosphate (FdUMP). The FdUMP could form a stable complex with thymidylate synthase (TS) in the presence of methylene tetrahydrofolate reductase (MTHFR) and then inhibits the production of deoxythymidine monophosphate (dTMP). While thymidylate synthase is inhibited, the cancer cells which reliant on the *de novo* thymidylate pathway undergo thymineless death (Houghton et al., 1997). Because dTMP is essential for DNA repair and replication, its depletion therefore causes cytotoxicity (Zhang et al., 2008). In clinical application, the combination of the folate analog Leucovorin and 5-FU can promote the clinical efficacy because it can promote thymidylate synthase ternary complex formation (Wolmark et al., 1999).

## Resistance in Fluorinated Pyrimidines

There are many defects of 5-FU, including systemic toxicities because of non-specific cytotoxicity for tumor cell, loss of efficiency due to poor distribution to tumor sites, and severely limited efficacy because of drug resistance (Alvarez et al., 2012). The serious systemic toxicities of 5-FU are commonly seen in gastrointestinal and hematopoietic effects (Gmeiner, 2020).

**FIGURE 2:**
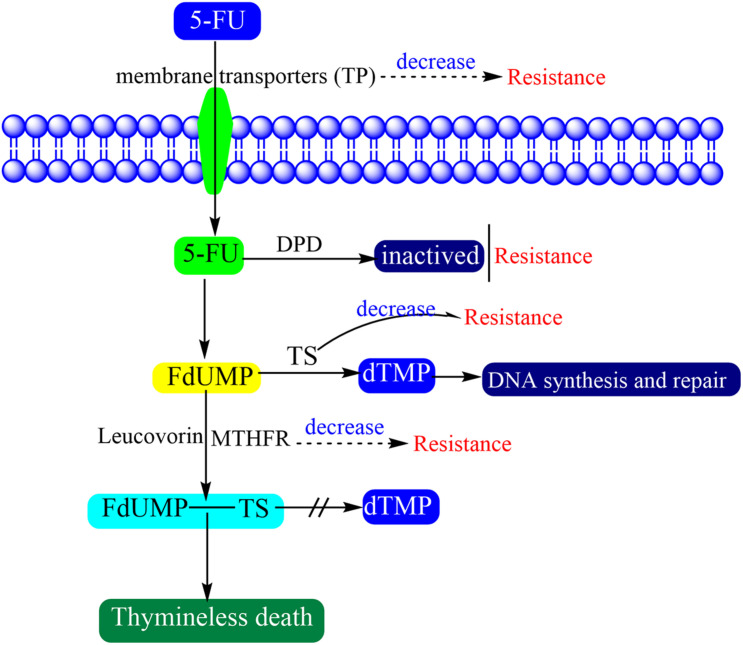
Schematic representation of drug effect and resistance of 5-FU.

There are multiple factors that may be responsible for 5-FU resistance (Figure 2) (Zhang et al., 2008). Antitumor drug resistance usually concentrates on alteration of drug influx and efflux, enhancement of drug deactivation, and mutation of the drug target (Longley and Johnston, 2005). The factors which affect the drug transition would affect the activation of 5-FU. 5-FU and other nucleic acid dugs present cytotoxicity only when pass through the cell membranes. However, the water-soluble character of 5-FU makes it so that it cannot pass through cell membranes by diffusion. Therefore, the specific nucleic acid membrane transporters are needed to help cells to absorb these drugs (Kong et al., 2004). Thymidine phosphatase (TP) is the main form of pyrimidine nucleoside phosphatase in humans, which helps cells to survive, promotes angiogenesis and inhibits apoptosis (Toi et al., 2005). When tumors present high levels of TP expression, they show more sensitivity to 5-FU (Soong et al., 2008).

Some enzymes are involved in the conversion and activation of 5-FU. DPD is an initial and rate-limiting factor in the catabolism of uracil and thymine which mediates the conversion of 5-FU to dihydrofluorouracil (DHFU) (Heggie et al., 1987; Zhang et al., 2008). The 5-FU resistance will generate while DPD activity increases in cancer patients, because the 5-FU will be converted to non-pharmacologically active metabolites before activation (Lu et al., 1993; Reti et al., 2010). TS is a key enzyme that catalyzes the conversion of dUMP to dTMP and is extremely important for DNA synthesis and repair. The TS loss will hamper cell proliferation and result in cell death (Costi et al., 2005). TS is overexpressed in most tumors and its high expression is an important factor of 5-FU resistance (Longley et al., 2003). MTHFR participate the conversion of 5-FU to a stable ternary complex which results in TS inhibition. The decrease in MTHFR activity finally inhibits the formation and stabilization of the ternary complex. Therefore, patients with a mutant genotype associated with decreased MTHFR activity are more sensitive to 5-FU (Lurje et al., 2008).

Autophagy and many signaling pathways also affect antitumor activity of 5-FU. Previous study has showed that inhibiting autophagy activity could enhance antitumor activity of 5-FU in colorectal cancers (Sasaki et al., 2012). Many signaling pathways involved 5-FU resistance, including Hippo/YAP, Wnt/β-catenin, Notch signaling pathway, Hedgehog, NF-kB signaling pathway, and so on (Xie et al., 2020).

To overcome the shortcomings of 5-FU, many fluorinated pyrimidines have been synthesized and are under biological evaluation. However, the DPD-inhibiting oral fluoropyrimidines such as eniluracil and 5-chloro-2,4-dihydroxypyridine (CDHP) have failed to improve outcomes for patients with metastatic colorectal cancer (Schmoll, 2003). The DPD inhibitors had combined with orally bioavailable fluorinated pyrimidine such as capecitabine or tegafur to verify the similar effect to continuous intra-venous infusion of 5-FU and did not prove molecules advantageous to continuous intra-venous infusion (Kobayakawa and Kojima, 2011; Aguado et al., 2014). Furthermore, more oral fluoropyrimidine such as S1 (tegafur/gimeracil/oteracil) are in progress phase I clinical trials for tumors, including EC (Ajani et al., 2020; Hosoda et al., 2020). However, up to date, the 5-FU is still the widest used fluorinated pyrimidine for cancer treatment. Many adjuvant drugs to promote activity of 5-FU, such as Non-steroidal anti-inflammatory drugs (NSAIDs), chloroquine (CQ), disulfiram, vitamin D analogs (VDAs), and Ca^2+^-activated G protein-coupled calcium-sensitive receptor (CaSR) (Xie et al., 2020). However, these agents are far from application in clinical practice.

## Taxanes (Paclitaxel or Docetaxel)

Taxanes are naturally occurring compounds and belongs to genus of Taxus. Paclitaxel (PTX) is a key member of taxane family and is a semisynthetic plant alkaloid that restabilizes the microtubule cytoskeleton against depolymerization (Tan et al., 2006). PTX has been widely used to treat a number of cancers and has been considered as a successful antitumor agent since it was first approved for the treatment of ovarian cancer in 1992 (Zhu and Chen, 2019). Docetaxel (DTX) is another widely used taxane for antitumor therapy, which was first found in 1990 (Sohail et al., 2018). PTX is a tricyclic diterpenoid compound and has a chemical formula of C_47_H_51_NO_41_.

Microtubules are the key members of the cytoskeleton, which play a notable role in various biological processes including maintenance of cell shape, molecular signaling pathways, conducing to the transportation of cell organelles, and production of the mitotic spindle to ensure the progression of the cell cycle (Jordan and Wilson, 2004; Magnani et al., 2009). PTX has a different antitumor action compared with other common tubulin-binding antitumor drugs which exert antitumor activity through impeding the assembly of tubulin into microtubules. It has a distinctive antitumor function which enhances the assembly of tubulin into microtubules and the production of non-functional microtubules (Weaver, 2014; Lee and Tan, 2018). In the physiological condition, there is a balance in the process of entering and eliminating tubulin proteins from microtubules. Upon effects of PTX or DTX, this balance is interrupted and the microtubules receive a stabilized form (Yvon et al., 1999). This results in cell cycle arrest, inhibition of mitosis, inhibiting growth and proliferation of cancer cells and apoptotic cell death (Weaver, 2014; Ashrafizadeh et al., 2020b).

## Resistance in Taxanes

Although the taxanes are highly active cytotoxic antitumor drugs, the serious adverse drug reactions and emergence of drug resistance still affect the clinical application.

The effects of antitumor activity could enhance in relation to drug dose increasing, however, the dose increasing not only promotes the drug’s effectiveness but also increases cytotoxicity (Ashrafizadeh et al., 2020a). The common toxicities of the drugs manifests in hair loss, hypersensitivity reactions, hematological toxicity (principally neutropenia), arthralgia, myalgias, and peripheral neuropathy (Markman, 2003). Fortunately, with appropriate management, both PTX and DTX generate easily treatable side effects and it would be a rare patient who is unable to proceed with taxane treatment due to unacceptable toxicity (Markman, 2003; Ashrafizadeh et al., 2020b).

A lot of molecular pathways and mechanisms are involved in the taxane resistance of cancer cells. Among the molecular mechanisms, autophagy is the most widely known mechanism related to PTX resistance. A study showed that the inhibition of autophagy can enhance antitumor activity of PTX. The self-digestion mechanism can stimulate cancer cells resistance into PTX chemotherapy. In this progress, non-coding RNAs is the most common molecular pathway that dually mediate or inhibit PTX resistance (Datta et al., 2019; Yang et al., 2020). Integrin subunit α2 (ITGA2) is an oncogene factor which promotes metastasis and growth ability of cancer cells and it is thought to reduce sensitivity of cancer cells of PTX through activation of Akt/FoxO1 signaling pathway (Ma et al., 2020; Qin et al., 2020). Furthermore, high mobility group box 1 (HMGB1) can activate c-Myc oncogene to induce PTX resistance (Jung et al., 2020; Lei et al., 2020).

To overcome the drug resistance and dose-limiting side effects, many studies have been proceeding. Many plant derived natural compound such as nobiletin (Feng et al., 2020), quercetin (Li et al., 2018), and resveratrol (Ozturk et al., 2019) have been used to inhibit PTX resistance and increase its antitumor activity in cancer cells. Irinotecan is a topoisomerase I inhibitor and proven to have benefits of survival and quality of life in 5-FU refractory colorectal cancer (Liu et al., 2020). Previous studies also considered it as a second- or third-line chemotherapy for EC which were refractory to platinum plus fluoropyrimidine chemotherapy (Wang et al., 2016).

For both EAC and ESCC, either adjuvant or neoadjuvant chemotherapy was proven to be superior to surgery alone in overall survival (Ando et al., 2003; Ychou et al., 2011). For the pathological node positive EC which received neoadjuvant chemotherapy or chemoradiotherapy, adjuvant chemotherapy showed a survival benefit compared to those that did not receive chemotherapy after esophagectomy (Samson et al., 2018). Platinum plus fluoropyrimidines (FP) or taxanes are an essential chemotherapy regimen for EC. Moreover, radiotherapy with FP regiment followed by surgery also showed superior overall survival compared with surgery alone (Tepper et al., 2008). Taxanes are also frequently used in chemotherapy for EC as a second-line chemotherapy for recurrent and metastasis EC. Many clinical trials indicate that paclitaxel and docetaxel are effective for both ESCC and EAC in extending median survival time (Muro et al., 2004; Ford et al., 2014). However, chemotherapy agents are exhibiting considerable cytotoxic effects including normal cells and the dose-limiting side effects restrict the chemotherapy’s effectiveness. Furthermore, the emergence of drug resistance is still the challenge for clinicians and drug developers and the effective approach to eliminate drug resistance and side effects has not been proven (Xie et al., 2020).

To overcome these limited therapeutic options and promote overall survival and quality of life, the personalized targeted therapies are designed based on the molecular characterization of EC (Maeda and Ando, 2019).

## Molecular Targeted Therapy

Despite the EC being mostly categorized into ESCC and EAC, the treatment options are largely similar both in the different histological types (Lagergren et al., 2017). However, the cellular and molecular data suggest that different histological types represent different genomic characterization. ESCCs closely resemble head and neck cancers while EACs more resemble gastric cancers (Cancer Genome Atlas Research Network, Analysis Working Group, Asan University, BC Cancer Agency, Brigham and Women’s Hospital, Broad Institute, et al., 2017).

The molecular characterization of EAC is divided into four etiological/genetic subtypes based on gastric adenocarcinoma molecular characterization classification (Cancer Genome Atlas Research Network, 2014): (1) EBV-associated tumors; (2) Microsatellite instability (MSI) tumors commonly with PIK3CA, EGFR and human epidermal growth factor receptor 2 (HER2) mutations; (3) Genomically stable tumors, (4) Chromosomally instability (CIN) tumors with TP53 mutations as well as RTK/RAS, VEGFR, and p110 amplifications (Barsouk et al., 2019). In these genomic subtypes, the MSI-high and EBV subtypes have shown great responsiveness to immune checkpoint inhibitors (ICIs), such as pembrolizumab (Kim et al., 2018; Le et al., 2018; Shitara et al., 2018). Only the transtuzumab have positive treatment responses in the CIN subtype and the other targeted therapies such as the cMET inhibitor, FGFR inhibitor, PARP inhibitor have failed (Shah et al., 2016; Bang et al., 2017; Van Cutsem et al., 2017). Although the EGFR tyrosine kinase inhibitors gefitinib did not meet significant overall survival benefits compared with a placebo in unselected EC patients, partial patients achieved disease control and benefits in progression-free survival (Dutton et al., 2014; Petty et al., 2017).

Despite the ESCC and EAC being similar to the responsiveness of chemotherapeutic agents, ESCC is significantly distinct from EAC at the genomic level (Ilson and Van Hillegersberg, 2018). However, the differentiated epigenetic alterations of growth advantage in ESCC are still not elucidated (Kang et al., 2015). The ESCC genomic characterization are divided into three molecular subtypes by The Cancer Genome Atlas Research Network: (1) the alteration in NrF2 pathway, which regulates adaptation to oxidative stressors including some carcinogens and some chemotherapy agents; (2) higher rates of mutation of NOTCH1 or ZNF750, more frequent inactivating alterations of KDM6A and KDM2D, CDK6 amplification, and inactivation of PTEN or PIK3R1; (3) No evidence for genetic deregulation of the cell cycle and only a few of TP53 mutations (Cancer Genome Atlas Research Network, Analysis Working Group, Asan University, BC Cancer Agency, Brigham and Women’s Hospital, Broad Institute, et al., 2017). Unfortunately, only a few cancer drivers are targetable and the druggability of a target is still a research question. The most approved targeted drugs in clinic are directed against kinases and some of them have been used to treat ESCC (Kang et al., 2015).

**FIGURE 3:**
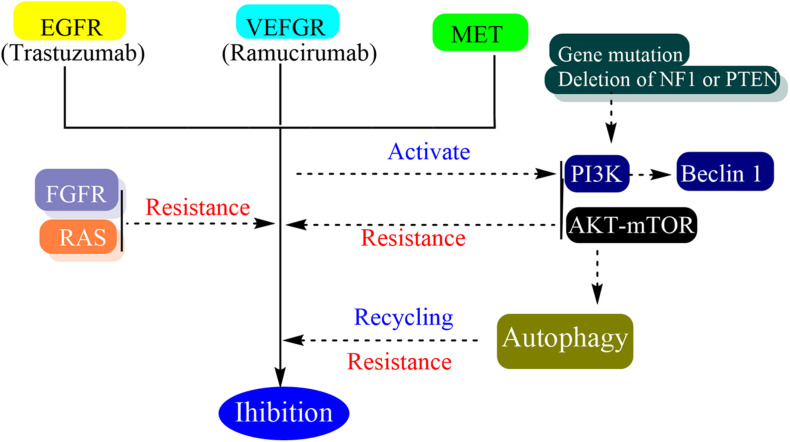
Schematic representation of drug effect and resistance of targeted therapy.

Many oncogenes and tumor suppressor genes have been revealed as promising targets for targeted therapy and immunotherapy. These agents usually inhibit the vascular endothelial growth factor (VEGF) signaling or receptor tyrosine kinases (RTK), such as the EGFR, erb-b2 receptor tyrosine kinase 2 (ERBB2 or HER2), and MET (*N*-methyl-N0-nitroso-guanidine human osteosarcoma transforming gene) (Figure 3) (Ilson and Van Hillegersberg, 2018).

### EGFR Inhibition

It is estimated that about 15–20% of advanced gastric and gastroesophageal junction (GEJ) cancers have overexpression or amplification of HER2 (Van Cutsem et al., 2015) and 12–41% in ESCC cases (Gonzaga et al., 2012; Zhan et al., 2012).

Cetuximab, as an anti-EGFR monoclonal chimeric antibody, is proven useful for head and neck cancers and colorectal cancer with the wild-type RAS gene. A phase 2 clinical study showed that cetuximab can be safely combined with standard chemotherapy and may increase the overall response rate, median progression-free survival, and median overall survival in advanced ESCC (Lorenzen et al., 2009). Moreover, another phase 2 clinical trial showed benefits of the addition of cetuximab to standard preoperative chemoradiotherapy in locally advanced ESCC (Brenner et al., 2019). However, it did not show additional benefit compared to chemotherapy alone for advanced gastric cancer (Lordick et al., 2013).

Gefitinib is EGFR tyrosine kinase inhibitor and a phase 3 trial did not show overall survival benefits for previous treated advanced EC including squamous-cell cancers (Dutton et al., 2014).

Lapatinib is a dual EGFR and HER2 tyrosine kinase domain. Some studies showed that the lapatinib combined with chemotherapy were efficacious for advanced ESCC (Reynolds et al., 2017; Guo et al., 2018). However, these studies are just in laboratory and phase 1 studies and it is far from being verified in patients.

Nimotuzumab is a recombinant humanized monoclonal antibody. It inhibits the EGFR-dependent intracellular signaling pathway via binding to the extracellular domain of EGFR (Hirano and Kato, 2019). Many phase 2 clinical trials and retrospective studies had showed that in combination of nimotuzumab to chemotherapy or chemoradiotherapy could achieve effective benefits for advanced and metastatic ESCC (Saumell et al., 2017; Jing et al., 2019). Pertuzumab is a humanized monoclonal HER2-targeted antibody which binds to a different epitope on the HER2 receptor protein than trastuzumab (Shitara et al., 2020). However, a phase 3 trial (JACOB) showed that adding pertuzumab to trastuzumab and chemotherapy did not significantly improve overall survival in patients with previously untreated HER2-positive metastatic gastric or GEJ cancer (Shitara et al., 2020).

Panitumumab as an anti-EGFR antibody did not promote survival for first-line chemotherapy for gastroesophageal adenocarcinomas in a phase 3 trial (REAL3) (Waddell et al., 2013). Moreover, it did improve survival and substantially increased toxicity for non-resectable, advanced, or metastatic ESCC in a phase 3 trial (POWER) (Moehler et al., 2020).

Trastuzumab is a recombinant humanized monoclonal antibody directed against HER2. In a phase 3 trial (ToG A), It was approved for the first-line treatment combined with chemotherapy for the metastatic, gastric, or GEJ cancers which have HER2 overexpression (Bang et al., 2010). However, many patients were still progressing under this regimen (Li and Li, 2016) and there were no clinical benefits for further trastuzumab utilization while the disease progressed (Makiyama et al., 2018). Trastuzumab emtansine (T-DM1) is a microtubule inhibitor, which conjugate monoclonal antibody agent trastuzumab and cytotoxic agent emtansine (DM1). However, the clinical trial showed that it was not superior to taxane as the second-line therapy for the previous treatment HER2-positive locally advanced or metastatic gastric or GEJ adenocarcinoma (Thuss-Patience et al., 2017).

### VEGF Inhibition

VEGFR inhibitors are largely used in gastrointestinal cancer including gastric and GEJ adenocarcinomas (Rajabi and Mousa, 2017).

Bevacizumab is a monoclonal antibody targeting VEGF-A which is mediated by two tyrosine kinase receptors, VEGFR-1 and VEGFR-2. Although it is an efficient treatment for several malignancies in combination with chemotherapy including colorectal cancer and lung cancer, it was not proven superior in overall survival time compared to chemotherapy for advanced gastric or GEJ adenocaecinoma (AVAGAST) (Ohtsu et al., 2011).

Nintedanib is a multikinase inhibitor that potently inhibits VEGFR1-3, FGFR1-3, and PDGFRa/b. A phase 2 trial showed that nintedanib did not have significant benefits in disease progression of first-line chemotherapy or metastatic esophagogastric adenocarcinoma compared with VEGFR2 inhibition alone and it has no value for the further development of EC (Won et al., 2019).

Ramucirumab is a fully humanized monoclonal antibody and is designed to inhibit VEGFR-2 via ligand binding which prevents VEGF ligands binding to VEGFR-2 epitopes (Spratlin et al., 2010). A double-blind, randomized phase 3 trial (RAINBOW) showed that there were significant benefits in ramucirumab plus paclitaxel group than compared to the paclitaxel group including the overall survival, progression-free survival, and therapy response rate (Wilke et al., 2014). For the patients who underwent first-line treatment with transtuzumab, paclitaxel plus ramucirumab is recommended as a second-line therapy regimen regardless of HER2 expression (Wilke et al., 2014). There are many other VEFG inhibitors in the clinical trial with no exciting results for gastric or GEJ adenocarcinoma, such as apatinib (Li et al., 2016), regorafenib (Pavlakis et al., 2016), lapatinib (Hecht et al., 2016) et al.

### MET Inhibition

MET is a proto-oncogene which encodes a receptor tyrosine kinase c-MET. Activation of c-MET is ordinarily indispensable to cell function such as morphogenesis, scattering and motility, cell proliferation, and protection from apoptosis (Mo and Liu, 2017). Rilotumumab is an inhibitor of the MET ligand hepatocyte growth factor. However, a phase 3 trial of chemotherapy plus rilotumumab as the first-line therapy for the advanced MET-positive gastric or GEJ cancer did not increase the overall survival time (Catenacci et al., 2017). Moreover, another phase 3 trial of the MET inhibitor onartuzumab which plus chemotherapy for HER2-negative and MET-positive advanced gastroesophageal adenocarcinoma also did not show significantly improved clinical benefits compared with chemotherapy (Shah et al., 2017).

Despite recent advances in genomic drivers of EC, there are only few targeted therapies available in clinical practice. Trastuzumab, ramucirumab, and pembrolizumab are the only three molecular therapy agents approved by the US Food and Drug Administration (FDA) for treatment of advanced and/or metastatic ECs (Fatehi Hassanabad et al., 2020).

## Resistance in Targeted Therapy

Although targeted therapy is better tolerated than traditional chemotherapy, it does produce toxicity based on several main mechanisms. The common side effects include hypertension, rash, diarrhea, hypothyroidism, proteinuria, depigmentation, and hepatotoxicity. Either antibodies or small molecular kinase inhibitors, the same target may have similar side effects. VEGFR kinase inhibitors usually cause hypertension and EGFR antibodies and kinase inhibitors usually cause rash (Liu and Kurzrock, 2014; Kang et al., 2015).

The intratumoral, intermetastatic, intrametastatic, or interpatient heterogeneities are related to various combinations of drivers and pathways. These heterogeneities may explain how the different patients have different treatment responses and even resistance under the same treatment and the favorable response patients initially could develop resistance over time (Kang et al., 2015). It is considered that the carcinogenesis is a successive new mutation which is driven by natural selection. Correspondingly, radiotherapy and targeted therapy may be the artificial selection which has a potent source to alter clonal dynamics. Therefore, the antitumor therapy may induce drug resistance (Greaves and Maley, 2012). In fact, targeted therapy is associated with a high rate of resistance at the beginning of the targeted therapy used in clinic (Kang et al., 2015). Patients will always develop resistance during this treatment (Merz et al., 2020).

Autophagy can contribute to drug resistance because autophagy contributes to cell homeostasis by eliminating damaged organelles and its activation can mitigate metabolic, oxidative, and endoplasmic reticulum stresses (Das et al., 2012). In the process of targeted therapy resistance, autophagy has been shown to play an important role in many different cancer types (Mizushima, 2018). Autophagy is involved in the recycling of some receptors which finally reduce the efficacy of targeted therapy and the cells which are deficient in autophagy are more sensitive to target therapies (Janser et al., 2019). The targeted therapy can trigger autophagy by several mechanisms such as activation of Beclin 1 through class III PI3K, induction of oxidative and endoplasmic reticulum stress, and alteration of AKT-mTOR pathway (Vera-Ramirez et al., 2018). *In vitro*, trastuzumab has been shown to induce autophagy and the basal autophagy of cell line which present intrinsic or acquired resistance to trastuzumab are increased (Chen et al., 2016). Although autophagy inhibitors showed the effect of reverting the resistance and increasing drug affects *in vitro* and *vivo*, the different targeted therapy blocking different pathways in several cancer types would activate a similar response to promote drug resistance (Mele et al., 2020).

Besides autophagy, many other resistance mechanisms have been identified including alteration of the drug target, alterations in upstream and downstream effectors resulting in pathway reactivation and bypass mechanisms (Groenendijk and Bernards, 2014). The activation of the PI3K/AKT/mTOR pathway is one of the bypass pathways to targeted therapy resistance. The increased PI3K signaling can be due to loss of function through gene mutations or deletion of NF1 or PTEN, or the activation of a wide range RTKs, EGFR, FGFR1, PGFR-β etc. (Zuo et al., 2018). Moreover, the interaction between tumor cells and microenvironment is also an important factor in drug resistance (Ahmed and Haass, 2018; Mourah et al., 2020).

After a certain period of trastuzumab treatment, almost all patients will develop drug resistance even if the primary resistance is very rare in patients in HER2 overexpression. The common mechanism of trastuzumab resistance is that the selection of HER2 will not amplify clones (Piro et al., 2018; Cavaliere et al., 2019). There are many mechanisms of primary or acquired resistance to anti-HER2 therapies, including impaired drug binding to HER2, constitutive activation of signaling pathways parallel or downstream of HER2, metabolic reprogramming or reduced immune system activation. However, only a few of them have been validated in clinical series (Vernieri et al., 2019).

Fibroblast growth factor receptor (FGFR) pathway activation was considered to be one of the mechanisms related to various targeted therapeutic resistance including EGFR TKIs (Luo et al., 2018). The FGF family has four tyrosine kinase receptors and is involved in cellular growth and tumor angiogenesis (Mossahebi-Mohammadi et al., 2020). FGF9 has a unique affinity to FGFR3 and the overexpression of FGFR3 may be responsible for transtuzumab resistance (Hecht et al., 1995; Touat et al., 2015). Bemarituzumab is a humanized monoclonal antibody specific to the human FGFR2b receptor. It demonstrated monotherapy clinical activity in the overexpression of FDFR2b late-line gastric cancer. However, there is no phase 3 trial to verify its effectiveness (Catenacci et al., 2019). Dovitinib is a selective FGFR3 inhibitor. While the trastuzumab-resistant murine models were treated with it, the tumor burdens were more reduced and the overall survival rates were longer than the control group (Piro et al., 2016). Pemigatinib is a selective, potent, oral inhibitor of FGFR1, 2, and 3. A phase 2 trial which intended to assess the safety and activity of pemigatinib in HER2 trastuzumab-resistant gastric or GEJ cancer showed that trastuzumab-resistance may benefit from the utilization of pemigatinib (Merz et al., 2020).

Additional mutations in receptor tyrosine kinases, RAS, and PI3K pathways appear to the mechanism of both intrinsic and acquired resistance to HER2 inhibitor. For patients with these co-alterations, there is a lower benefit from trastuzumab treatment and it has a shorter progression-free survival (Janjigian et al., 2018). The combination with anti-angiogenesis trastuzumab and anti-EGFR ramucirumab has a good experience in durations of response in esophagogastric cancer. It is considered that the resistance inducing by angiogenesis and HER2 signaling may be reversed with the inhibition of the angiogenesis pathway. However, the bevacizumab combined with cetuximab or panitumumab received disappointing results in the CAIRO-2 and PACCE studies (Hecht et al., 2009; Tol et al., 2009). Furthermore, the combination with HER2 inhibitor and VEGFR inhibitor were associated with higher rates of side effects, even a detrimental effect on survival.

In a short, although molecular-targeted therapy is widely used in other solid tumors such as breast, leukemia, colorectal, lung, and ovarian cancers (Lee et al., 2018), it has limited benefit in EC treatment because of intrinsic resistance or acquired resistance.

## Immunotherapy

Immunotherapy has entered a new era in cancer treatment (Dong et al., 2018; Peinemann et al., 2019; Zhao and Huang, 2020; Zhu et al., 2020). A lot of immune modulatory strategies have been developed in the past few years, including ICIs, adoptive cellular therapy, cancer vaccines, oncolytic viruses, and so on. Among these, ICIs have greatly impacted on routine clinical practice which has achieved unprecedented results in many tumors (Dong et al., 2018; Ribas and Wolchok, 2018).

Programmed cell death-1 (PD-1) was first identified in 1992 and also called CD279 (Ishida et al., 1992). PD-1 is a 55-kDa transmembrane protein with 288 amino acids and a member of the B7-CD28 family of cell surface receptors including CTLA-4, CD28, BTLA, and ICOS, which is expressed on activated T cells, B cells, NKT cells, monocytes and macrophages (Sharpe and Freeman, 2002; Francisco et al., 2010). While PD-1 is binding to its ligands, the active immune cells are inhibited. The PD-L1 (CD279 and B7-H1) and PD-L2 (CD273 and B7-DC) are the two recognized ligands of PD-1, which belong to the B7 family (Sanmamed and Chen, 2014). Although PD-L1 and PD-L2 is different from the expression pattern, both of them bind to the same receptor and inhibit immune response, therefore they do not have any different roles (Momtaz and Postow, 2014). PD-L1 is constitutively expressed on the surface of immune cells, professional antigen-presenting cells (APCs) that are mainly defined by expressing MHC class 2, such as macrophages, B cells and dendritic cells (DCs), and costimulatory molecules to CD4^+^ T cells. PD-L1 is also expressed on the surface of non-professional APCs which present antigen to cytotoxic CD8^+^ T cells (Francisco et al., 2010).

**FIGURE 4:**
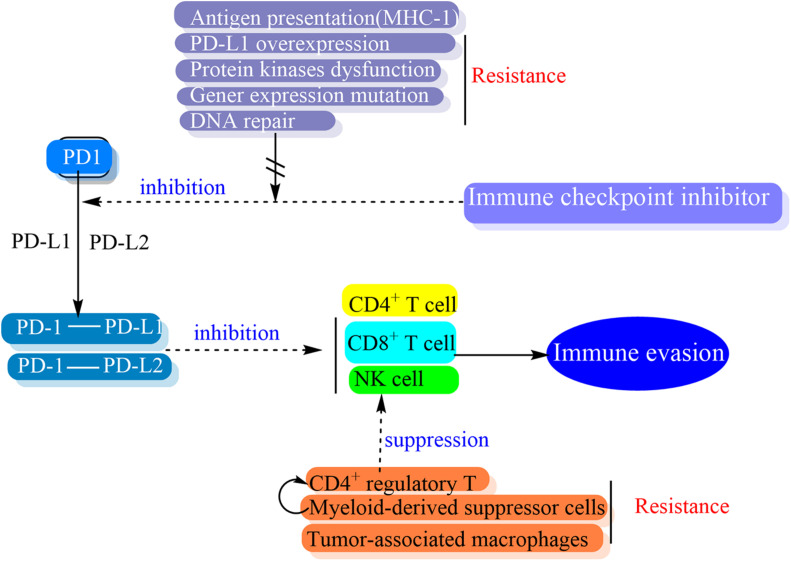
Schematic representation of drug effect and resistance of immune checkpoint inhibitor.

The PD-1/PD-L1 pathway has a role in promoting mechanisms of tumor immune evasion. This is the theoretical basis of the development of monoclonal antibodies targeting either PD-1 or PD-L1. The block of the binding of PD-1 and its ligands has the potential to make tumor cells exposure to the immune regulatory activity of effector T cells and resume an effective antitumor immune response (Figure 4) (Sharpe and Freeman, 2002; Francisco et al., 2010; Pardoll, 2012).

The efficiency of the PD-1/PD-L1 inhibitors is associated with the PD-L1 expression and/or tumor mutation burden (TMB) in tumor cells (Lawrence et al., 2013). For EC, about 14.5–82.8% patients have tumors with PD-L1 expression and high TMB (Hong and Ding, 2019). The pembrolizumab and nivolumab are the recommended anti-PD-1 monoclonal antibodies based on several clinical trials for EC either ESCC or EAC (Hong and Ding, 2019).

Pembrolizumab is a fully humanized IgG4-k monoclonal antibody which is potent and highly selective against PD-1 (Wolchok, 2015). In many clinical trials, pembrolizumab demonstrated manageable toxicity and durable antitumor activity in patients with previous standard therapy failed, PD-L1–positive advanced, recurrent, and metastatic EC (Doi et al., 2018; Fuchs et al., 2018; Kojima et al., 2019; Shah et al., 2019). Most of the patient in these trials who achieved overall survival superiority had an ESCC and PD-L1 combined positive score (CPS) of more than 10 (Kojima et al., 2019). Based on these clinical trials, pembrolizumab as a second-line therapy has been approved by FDA for advanced ESCC patients with PD-L1 CPS ≥ 10 (Yamamoto and Kato, 2020). Moreover, many clinical trials had combined pembrolizumab and trastuzumab (HER2 inhibitor) plus standard first-line chemotherapy for HER2-positive advanced and metastatic gastroesophageal cancer and the results were safe and tolerable (Bang et al., 2010; Tabernero et al., 2019; Janjigian et al., 2020). The benefits were observed when adding trastuzumab and pertuzumab to neochemoradiotherapy for resectable HER2-positive EAC (Stroes et al., 2020).

Nivolumab is a high-affinity, humanized IgG4 monoclonal PD-1 antibody (Wolchok, 2015). Many clinical trials showed that nivolumab had benefits in improving the overall survival of metastatic renal cell carcinoma (Peinemann et al., 2019). A phase 2 trial (ATTRACTION-01) showed that nivolumab had significant efficacy and safety for metastatic or recurrent ESCC (Kudo et al., 2017). The phase 3 trial (ATTRACTION-3) showed superiority of nivolumab for the second-line treatment of all ESCC patients regardless of PD-L1 expression (Kato et al., 2019; Kojima et al., 2019). Thus, nivolumab has approved by the FDA as a monotherapy for advanced ESCC patients after prior standard chemotherapy regardless of PD-L1 expression (Yamamoto and Kato, 2020).

There are many clinical trials in progress for other anti-PD-1 antibodies, such as tislelizumab (Xu et al., 2020), atezolizumab (Bando et al., 2020), durvalumab (Mamdani et al., 2019), and so on. However, most of them are under phase 1/2 clinical trials and far from clinical application.

In short, for metastatic or recurrent ESCC patients, ICIs such as pembrolizumab and nivolumab are standard treatment for second-line chemotherapy (Kato et al., 2019; Kojima et al., 2019). However, there is little evidence for the efficacy and safety of ICIs for metastatic or recurrent EAC patients (Yamamoto and Kato, 2020).

## Resistance in Immunotherapy

There are still many questions and important challenges to conquer in ICIs application (Akin Telli et al., 2020). Firstly, although the immunotherapy has desirable antitumor effects, the inhibition of immune checkpoints may also result in loss of peripheral tolerance and subsequently arouse immune activation on non-tumor cells, which finally causes unintended tissue damage and represents multisystem organ dysfunction. Almost every organ system could manifest as tissue damage, in which the dermatologic, gastrointestinal, endocrine, and pulmonary systems are the most commonly affected (Hryniewicki et al., 2018). In previous studies, the most common immune-related adverse events (irAEs) of PD-1 inhibitors (pembrolizumab and nivolumab) were fatigue, pruritus, rash, and diarrhea. Furthermore, most of them (more than 95%) are low grade adverse effects. Secondly, compared with molecular targeted drugs, the ICI drug is more prone to the occurrence of drug resistance (Lim and Rizos, 2018). The drug resistance of ICIs are also divided into intrinsic and acquired resistance (Sharma et al., 2017). Previous studies showed that most tumors rarely exceed 40% remission rates and most of them were partial response (Sharma and Allison, 2015). Furthermore, the resistance to anti-PD-1/PD-L1 therapy was up to 70% of patients and the primary resistance was up to 60% (O’donnell et al., 2017; Nowicki et al., 2018). In the ATTRACTION-3 trial, the treatment responses between nivolumab and chemotherapy groups for ECs were similar (19% vs. 22%) and the responses to immunotherapy needed more time to be apparent compared with chemotherapy. However, the durability of the response in the nivolumab group was longer than that in chemotherapy groups. Furthermore, the proportion of patients with progressive disease were higher in the nivolumab group than that in chemotherapy group. Based on this, we could think that the immunotherapy drug nivolumab may be as beneficial as chemotherapy, but the intrinsic resistance and acquired resistance remain major obstacles to immunotherapy of EC (Kato et al., 2019).

The mechanism of ICIs resistance has not been fully elucidated. One cause of it may be related to the heterogeneity of the tumor process and the essence of it is the tumor immune escape. Generally, the drug resistance of ICIs is considered to involve in the interaction of internal and external factors between tumor cells and immune cells within the tumor and the interaction may present a dynamic multilevel change process (Wang and Wu, 2020). The tumor-cell related factors of resistance are considered as intrinsic mechanisms while the immune-cell and micro-environment related factors refer to extrinsic mechanisms of resistance (Pitt et al., 2016). A variety of intrinsic and extrinsic factors are involved in tumor cell evasion from immune activation at the genetic, enzymatic, and cellular levels, such as the regulation of TME, intracellular protein mutations, oncogene signal transduction pathways, epigenetic changes, etc. (Wang and Wu, 2020). Whether intrinsic or extrinsic factors, the mechanisms of resistance are concentrated on antigen presentation, cytotoxic T-cell activation and trafficking, as well as the stimulation of the immune-inhibitory axis (Haibe et al., 2020).

There are many cancer cell alteration processes related to intrinsic resistance. One of them is the alteration of antigen presentation which affects the immune recognition. The surface of tumor cells often present loss or down-regulation of the major histocompatibility complex class I molecules (MHC-1), and this is suggested to be a mechanism of immune escape of the tumor (Baba et al., 2007). Every tumor type has a different escape mechanism (Yoo et al., 2019) and various tumors demonstrate deregulated expression of MHC-1 (Garrido et al., 2017). The frequency of MHC-1 loss with concurrent PD-L1 expression was approximately 12.2% in ESCC (Ito et al., 2016). The alteration of MHC-1 transcription and expression will impact on anti-PD-1/PD-L1 therapies (García-Aranda and Redondo, 2019). The cell signaling also affects immune response. The abnormal expression or dysregulation of protein kinases is involved in different hallmarks of cancer including survival, motility, metabolism, angiogenesis, proliferation, resistance to standard therapies, and immunotherapies and escape of antitumor immune responses (Gross et al., 2015; Garcia-Aranda and Redondo, 2017). PD-L1 overexpression can be a major hinderance for anti-PD-L1 therapy via overexpression tumor specific T-cells and apart from its immunosuppressive function (García-Aranda and Redondo, 2019). Besides the role of protein kinases on the expression of both PD-1 and its ligands, the dysregulation of protein kinase pathways is also a main cause of apoptosis resistance against immune response (Garcia-Aranda et al., 2018). The absence of tumor neoantigen expression and presentation leads to a lack of recognition by T-cells and results in the inability of tumors to respond to PD-1/PD-L1 inhibitor therapy (Bai J. et al., 2017), moreover, other alterations such as apoptosis suppression or DNA repair promoted are also associated with treatment resistance (Mansoori et al., 2017). The anti-apoptotic activity of altered protein kinases is tightly related to tumor resistance against most therapeutic treatments including immunotherapies (Garcia-Aranda and Redondo, 2017). Either the alterations of receptor kinases in their activity, abundance, cellular distribution and/or regulation can affect the functioning of signal transduction routes and result in the constitutive activation of downstream kinases like PI3K/AKT, MAPK, EGFR, or JAK/STAT (Garcia-Aranda and Redondo, 2019). Furthermore, the alterations also affect different transcription factors which command cell survival and trigger PD-L1 expression (Bai J. et al., 2017).

There are many gene expression mutations which affect immune response (Trujillo et al., 2019). Loss of B2M and defective IFN-γ signaling are tightly associated with T cell-resistant phenotype and tumor-intrinsic determinants of resistance to immunotherapies. The B2M is the subunit necessary for antigen presentation by MHC-1 and the tumor resistant anti-CTLA-4 or anti-PD-1 therapy no longer express B2M (Sade-Feldman et al., 2017). Defective IFN-γ signaling, such as through inactivating mutations in Janus kinases (JAK1 or JAK2) or in the interferon-gamma receptor 1 (IFNGR1), has also been suggested to relate to resistance of anti-PD-1 therapy (Sucker et al., 2017). WNT signaling has not only been involved in tumor progress but also in the ability of tumor cells escaping from different types of cellular stresses, including drug treatments and host immune response (Martin-Orozco et al., 2019). The tumor cell-intrinsic activation of the Wnt/β-catenin pathway mediate T cell exclusion from the tumor microenvironment and it lead to primary resistance to ICI therapy. Up-regulation of β-catenin by cancer cells could cause tumor recurrence and result in secondary resistance to immunotherapy (Spranger et al., 2015).

The DNA damage responses also affect the treatment benefits. Many studies suggest that DNA repair plays an important role in driving sensitivity and response to ICIs (Mouw et al., 2017). Many reports have manifested that a specific DNA damage exposure or a specific DNA repair pathway deficiency is related to ICI response. Loss of normal DNA repair fidelity may lead to an increase in mutational burden and ICI response of tumors (Rizvi et al., 2015).

The expression of different checkpoints to prevent T-cell activation leads to secondary resistance. Single antagonistic PD1/PDL1 pathway has limited function in improving immune cells and is prone to drug resistance (De Sousa Linhares et al., 2018). Besides the PD-1, a lot of high expression of immune inhibitory checkpoints are related to T-cell function including T cells immune globulin mucin-3(TIM-3), CTLA-4, Lymphocyte activation gene 3(LAG-3), B and T lymphocytes attenuation factor (BTLA), T-cell immune globulin and ITIM structure domain proteins (TIGIT) etc., and these checkpoints also affect the efficacy of PD-1/PD-L1 antibody (Curdy et al., 2019).

Alteration of T-cell activation would lead to extrinsic resistance (Shitara and Nishikawa, 2018). CD4^+^ regulatory T (Treg) cells is one of the most common immune-suppressive cells in tumors and a highly immune-suppressive subset of CD4^+^ T cells that maintain immune homeostasis. It can suppress antitumor immunity and promote cancer progression (Togashi and Nishikawa, 2017). *In vitro*, PD-1 upregulation by Treg cells increases the suppression of the CD8^+^ T cell response (Park et al., 2015). PD-1 expression modulates the activation threshold of T cells and maintains the balance between regulatory and effector T cells (Zhang et al., 2016).

Myeloid-derived suppressor cells (MDSC) partly mediated profound immunosuppression in a number of patients which do not respond to immunotherapy. The heterogeneous population of immature myeloid cells can significantly inhibit anti-tumor activities of T and NK cells and stimulate Treg cells, which leads to tumor progression (Weber et al., 2018). Activated MDSC express high levels of PD-L1 that interacts with PD-1 on T cells and causes their exhaustion (Dieterich et al., 2017). MDSC has been shown to drive the differentiation of CD4^+^ T cells into immunosuppressive Treg cells and reduce the anti-tumor activity of effector T cells (Pan et al., 2010).

Tumor-associated macrophages (TAM) represents immune suppressor cells in the solid tumors that restrict anti-tumor immune reaction induced by CD8^+^ T cells (Cassetta and Kitamura, 2018). While the TAM is abundant in a tumor microenvironment, TAM is proposed as one of the important therapeutic targets to promote the efficacy of immunotherapies utilizing checkpoint antagonists (Mantovani et al., 2017).

Pro-inflammatory cytokines such as Interferon-α, Interleukin-2, TNF-α, TGF-β et al. can contribute to cancer immunotherapy, acting on every phase of the cancer immunity cycle (Chen and Mellman, 2017). The cellular immunotherapies can be optimized by the incorporation of cytokine genes (Chmielewski and Abken, 2015). Therefore, cytokines can elevate antigen priming, increase the number of effector immune cells in the TME and promote their cytolytic activity. The ability to expand and reactivate effector NK and T lymphocytes, promote tumor infiltration, and persist in the TME make the cytokines important molecules to overcome resistance (Berraondo et al., 2019).

Although many mechanisms are assumed to be associated with immunotherapy resistance, it is difficult to get a universal biomarker for resisting drug resistance (Haibe et al., 2020; Wang and Wu, 2020). Similarly, the specific mechanisms of immunotherapy resistance in EC are also far from elucidated because the immunotherapy agents have not been widely used in clinical practice. Until now, only pembrolizumab and nivolumab are approved for second-line therapy for advanced/metastatic EC by the FDA. Sun et al. (2020) had reported that an advanced ESCC patient who received nivolumab therapy developed hyperprogressive disease shortly. They analyzed that the mechanism of hyperprogressive disease may be associated with PI3K/AKT signaling pathway, because the PI3K/Akt signaling pathway was activated in tumor progression patient (Sun et al., 2020). Wang et al. (2019) had evaluated the expression of LAG-3, CTLA-4, and the density of CD8^+^ tumor-infiltrating lymphocyte in resected ESCC. The results showed that positive LAG-3 expression was significantly correlated with positive CTLA-4 expression and poor prognosis in ESCC (Wang W. et al., 2019). The positive expression of LAG-3 and CTLA-4 may prevent T-cell activation and lead to secondary resistance. Zheng et al. (2020) reported that CD39 was overexpressed on NK cells from EC patients and the frequency of CD39^+^ NK cells correlated with the poor prognosis. Moreover, IL-6 can induce CD39 expression and CD39^+^ NK cells presented an exhausted phenotype (Zheng et al., 2020). Because the dysfunction of NK cells could lead to immune escape, the activated IL-6 may increase CD39 expression and the NK cells exhaustion could cause immunotherapy resistance. However, these phenomena should not elucidate the mechanisms of immunotherapy resistance for EC and the studies of resisting the immunotherapy resistance are still ongoing.

## In Summary

The pharmaceutical therapies are essential for EC. For the advanced EC, the neoadjuvant therapy including chemotherapy plus radiotherapy and/or immunotherapy is effective to achieve clinical benefit and even pathological complete response. For the recurrent, unresectable, and metastatic EC, the pharmaceutical therapy is a limited effective regimen to alleviate the disease and prolong the PFS and OS.

The regimen of platinum complexes plus fluorinated pyrimidines or taxanes is still the first-line therapy for EC. Although the targeted therapies are effective on other solid tumors such as non-small cell lung cancer, especially adenocarcinoma, the molecular targeted therapy for EC is not as effective as expected and only approved as a second-line therapy by the FDA. Furthermore, the targeted therapeutic effect in EAC is more effective compared with that in ESCC. Immunotherapy has entered a new era in cancer treatment. The ICIs are proven to provide a significant benefit in many malignant tumors compared to traditional therapy and will become the mainstream pharmaceutical therapy for the unresectable and progressive or metastatic EC. Compared to EAC, the ESCC receive more effective benefits from ICIs.

However, the significant issues of pharmaceutical treatment are still the dose-limiting side effects and primary or secondary drug resistance. Despite the fact that the mechanisms of drug resistance have achieved many profits, the dose-limiting side effects and drug resistance still restrain the clinical application and diminish the effectiveness of pharmaceutical treatment.

## Author Contributions

CM and XZ contributed to the conceptual idea, reviewed, analyzed the literature, and wrote the manuscript. XX and SL assisted in compiling background material. YuY and CZ edited the manuscript and provided the suggestions for the revision of the manuscript. YZ and YoY provided the conceptual idea, analyzed the literature, and reviewed and edited the manuscript. All the authors contributed to the article and approved the submitted version.

## Conflict of Interest

The authors declare that the research was conducted in the absence of any commercial or financial relationships that could be construed as a potential conflict of interest.
